# Mobility
1An Address given at a special meeting of the Bristol Medico-Chirurgical Society, held on May 20th, 1903.


**Published:** 1903-06

**Authors:** John Chiene

**Affiliations:** Professor of Surgery, University of Edinburgh.


The Bristol

Medico-Chirurgical Journal.

"*Scire est nescire, nisi id me*
*Scire alius sciret.*"

JUNE, 1903.

### MOBILITY.[1]

BY

JOHN CHIENE, C.B., LL.D., F.R.S.E., M.D.,
*Professor of Surgery, University of Edinburgh.*

THE kind invitation to spend an evening with you was gladly accepted, because I am a firm believer in intellectual communism. I trust that "I grow old learning things," and I am here to-night in the quest of further knowledge.

I trust that we are all book lovers, but if we are true to ourselves we will readily acknowledge that books are not everything. Books doubtless have their uses: books have also their limitations. The constant reliance on printed matter tends to onesidedness. Personal intercourse—wit sharpening wit—is, to my mind, the most important factor in true education. The kindergarten with its pleasures—the sugar-candy on Wednesdays—the absence of pain—is the essence of what is best in education. I am not prepared to go the length of a great

[1] An Address given at a special meeting of the Bristol Medico-Chirurgical Society, held on May 20th, 1903.

thinker, that a child is only to be taught what he wishes to learn, but I am of opinion that the essence of education is to find out what a child wishes to know, and teach him that. Knowledge is a mental ferment; as it grows it increases a desire for more knowledge.

I have been a teacher for a good many years, and each year I feel more and more the need of a resting time—resting, not in the sense of idleness, but in a change of work, the only true rest—a change of locality, a change of scene and of surroundings. I verily believe that a time will come when every teacher will be turned periodically adrift and compelled to wander, like the peripatetic philosophers, on the face of the earth, and speak to and learn from (he cannot do the one without the other) his neighbour, if possible to do good and certainly to get good from his neighbours, face to face, giving them his enchiridion, receiving from them their enchiridion. I use the word "neighbour" in the sense of the poor man whose good fortune it was to fall into the hands of the good Samaritan: that parable read in the widest sense surely means that we all require help—a helping hand. We have all fallen amongst thieves, such as Parochialism, with its foster-sisters, Self-sufficiency and Narrowness, in the main due, from the standpoint of the teacher, to constantly addressing an audience always changing as regards the individual, and always the same as regards the outlook—an audience whose main standpoint necessarily is the examination test, that mist which hampers teacher and taught. As the picture without atmosphere, so is the lecture with a limited horizon, bounded by the benches of the examination hall.

A visit such as this does me a world of good, takes me out of myself, dispels these thoughts, disarms these thieves, removes mental stagnation and psychical paresis: you are in very deed good Samaritans. I am deeply grateful, my message is one of thankfulness. However little I can say, may I say it reverently, knowing well that its very imperfection is its chief value, acting as a stimulus to my hearers.

Many changes have taken place since I gave my first course of lectures on surgery. I have tried to follow these changes, and have endeavoured to form a just conclusion as regards their benefits. I am not going to compare the surgery of 1870 with the surgery of to-day, nor can I even indicate my steps and stumbles, nor the men who have influenced me and inspired me—my heroes. May I in this city, however, say of one of my heroes how highly I esteemed the friendship of Greig Smith, and how deeply I mourned his early death, and the loss to surgery and to his friends;—another example of that ever-present thought that we often appear to lose those whom we think we can least afford to lose: their works remain after them, and they and we are taken in their and our good time. We take comfort that "great things done endure."

I am well aware that my presence here to-night is mainly due to the position which I hold in the medical school in the grey metropolis of the north; but still I also know that there is a personal element in your kind invitation, and I trust I will be excused if I say a word on my personal views regarding some important practical points in the borderland, where the work of the general surgeon closely approaches the work of the general practitioner. It is always well to study dying-out races, decaying species: the few remaining buffaloes in America are interesting, and well worthy of our keenest observation. If I can read the times, we, the general surgeons and the general practitioners, will soon be displaced from our pride of place. As I have sometimes said, the aurists and laryngologists working down from above, the gynæcologists working up from below, leave us stranded on the umbilicus, trying to eke out a bare existence on the scanty herbage, and thankful for the crumbs which fall from the rich man's table. Let me then state shortly some of my beliefs regarding an important group of cases which come immediately under the care of the general practitioner, and in which the general surgeon may be consulted; cases which, if care is not taken, may ultimately pass into the hands of those who are often called irregular—I prefer the term irresponsible—practitioners.

Let us consider together *recent subcutaneous injuries of Bones and Joints; Sprains, Dislocations, and Fractures*. I have chosen this subject because of its deep practical importance, and because at the present time, if I may be permitted to try and read the signs of the times, a great question has to be considered. Is rest the *summum bonum* in the treatment of these injuries? As far as I can judge, it is being discredited, and physiological movement is striving to take its place. Is it the case that stagnation of the venous and lymphatic flow in the injured part is the *great obstacle* to repair? Is it true that rest is an evil thing because it encourages this stagnation, that movement is a good thing because it removes this stagnation? Physiological activity can be induced by rubbing, and by voluntary muscular movements which both act by encouraging the blood and lymph-flow through the tissues. Passive movements are not in any sense, equivalent to, or to be compared to, voluntary movements. It is to these questions that I would ask your attention: about them there may be much difference of opinion, hence their value as a stimulus, as a fit subject for discussion.

How is a recent sprain—say, of the ankle joint—treated? Fomentations sprinkled with laudanum, and complete rest in bed, used to be in all, and still is in the hands of many, the orthodox treatment. After a time, varying with the severity of the accident, a starch bandage displaces the fomentation, the sofa takes the place of the bed. The result, as a rule, is a swollen, stiff, and often painful joint and a tedious convalescence. The fear was, and still is, inflammation; and in the background there was, and is, the dread of tubercle attacking the injured part: to avoid these evils, rest the joint.

We now know that inflammation, in the clinical sense, is not due to movement, but is due to organisms and their toxins; we also know that tubercle is due to a special bacillus; we also know that a free circulation through the injured part, instead of being an encouragment, is an obstacle to the deposit of organisms; the higher the physiological activity, the less the risk of organismal infection. We further know that the paresis of the blood-vessels, and the stagnation of the blood stream, and the extravascular effusion into the tissues, encourage, not only the primary deposit, but the secondary multiplication in the tissues. Sprains treated in the way described have been the happy hunting-ground of the bone-setter. The patient gets tired: he has kindly friends who advise him, and he slips away to the bone-setter, who, by a twist, breaks down an adhesion and restores him to sudden usefulness. It is not to this aspect of the question to which I to-night direct attention. I desire to speak of *recent injuries and their treatment.* I don 't speak of the evil results of too prolonged rest and their treatment. May I, however, say one word. Don't be afraid of movement in stiff joints of a chronic character, and of a traumatic origin. Break down adhesions, and after they are broken down don't let the patient rest and get stiff again. Find out the tender spot. Find out the movement that causes pain. Relax the tender tissue by position, and, fixing the painful spot with the thumb of one hand, suddenly perform that movement which will forcibly stretch, or tear, or break down the adhesion. Remember this, that a rotatory movement in ball and socket joints is most valuable; and, lastly, leave tubercular joints alone. Excuse this short digression. Let me return to the recent sprain, and indicate another line of treatment. (1) elastic pressure to prevent effusion, and to encourage the removal of any effusion which has taken place; (2) rubbing night and morning, begun at once; (3) voluntary movement as soon as possible: all these means are to be used with one object, namely, restoration as soon as possible, of a normal circulation of blood and lymph through the injured part; in other words, physiological activity. N.B.—The elastic pressure must be so used that there will be no interference with the circulation, which is the essential to rapid recovery. This method of dealing with sprains has many advocates. I have used it for twenty years, with one exception. Passive movement has had with me too prominent a place. I have only recently appreciated the value of voluntary movements, and recognised that passive movements are not an equivalent.

As with sprains so with *dislocations.* Prolonged rest is not now considered by many an essential after reduction; rubbing and gentle voluntary movements have taken its place. Care must, of course, be taken to avoid that movement which causes the head of the dislocated bone to press on that part of the capsule of the joint which gave way when the dislocation occurred. For example, we avoid flexion at the hip-joint in dislocation backwards of that part; we avoid abduction of the arm in dislocation at the shoulder-joint. Gentle voluntary movements in other directions are perfectly justifiable after a week of rubbing.

We now come to more debateable ground. Are we to apply the same principles to all *simple fractures?* Lucas-Championnière in France, and Wharton Hood in this country, emphatically say "Yes"; and I think that gradually their views are gaining ground. One cannot but wish that Lucas-Championnière's work[1] was more generally known to practitioners in this country: many less useful books have passed through the translator's hands. There can be no doubt that in 90 per cent. of the fractures which take place in Great Britain, the practitioner who has to treat them fears movement. My friend, Lucas-Championnière, says: "Le mouvement c'est la vie." I have for long held that as the ribs, which are never at rest, heal rapidly after fracture, we need not be so afraid of movement. It has also to be noted that, in my opinion, the best way to treat a transverse fracture immediately above the condyles of the humerus is by gently binding the arm in the fully flexed position to the constantly-moving chest-wall. Possibly the constant movement of the chest-wall is practically a form of rubbing. Certainly in neither case has the patient any discomfort from the gentle movement. I have not yet given up all splints: I may come to that as I gain experience. My view at present in the treatment of fractures is, no interference with the circulation through the injured part; gentle elastic pressure; appropriate splints to steady the part; splints so arranged that they can be easily removed for a daily rubbing. Begin with a gentle rubbing when you are first called to a case. This rubbing is applied as in sprains, beginning on the proximal side of the seat of injury and rubbing in the direction of the venous and lymphatic flow, gradually coming down over the seat of the injury. During the rubbing the fracture is steadied by the other hand of the rubber, or, if both hands of the rubber are used, an assistant must steady the fracture; and, lastly, very gentle voluntary movements are

[1] Traitement des Fractures par le Massage et la Mobilisation, Paris, 1895.

encouraged after the first week, the fracture being steadied during their performance. These voluntary movements are of infinitely more value than passive movements. I gladly welcome this distinction, because I have long taught that the act, say, of flexion, cannot be performed without an efficient extensor, in other words, both flexion and extension are necessary and essential. If I flex the forearm of another person, I do not call into play his flexors, nor do I ask the help of his extensors; if I flex my own forearm voluntarily, I contract my flexors and elongate my extensors; and *vice versâ* when I extend my forearm I contract my extensors and elongate my flexors. When I abduct my arm from my side I contract my abductors and elongate my adductors; when I adduct my arm to my side I contract my adductors and elongate my abductors. In voluntary movements I call into play a complicated nervous mechanism which takes no part in passive movements. When, for example, in a fracture of the humerus I passively flex my patient's forearm, the least pain is at once a message to the triceps to contract reflexly in order to prevent the painful movement. If, however, I ask my patient voluntarily and gently to flex his forearm, promising him that I will fix the fracture and support the forearm, confidently telling him that he need not fear the pain, then his extensor muscle takes an intelligent part in, and assists the action; it does not act as an opponent as in a similar movement performed passively.

I beg cordially to acknowledge the help that Championnière and Wharton Hood have been to me personally. If anyone reads Wharton Hood's[1] book on the *Treatment of Injuries*, it will be observed that he dislikes passive movements as much as he loves voluntary movements. Everything on the limb must be arranged with a double object: (1) that it can be easily removed for the daily rubbing, and for the regulated voluntary movements; (2) that it, when applied, has not the slightest effect upon the free circulation through the limb. Remember the value of a many-tailed bandage which can be easily removed, and the necessity of plenty of elastic wadding. Always leave the limb beyond uncovered, so that one will at once by its

[1] Wharton Hood, *Treatment of Injuries*, 1902.

appearance judge of the integrity of the circulation through the injured part.

Rubbing and regulated voluntary movements are, in my opinion, to be welcomed in all subcutaneous injuries—sprains, dislocations and fractures. I must add a word of warning. To allow a patient to move a sprained ankle voluntarily does not mean that you are to allow him to bear his weight upon the limb: with crutches he can do it gently and gradually; and, as he gets more and more confidence, he will increase the range of movement, and the amount of weight he bears on the limb. So in fractures of the lower extremity, he must not bear his full weight on the limb until union has taken place. During the consolidation of the fracture the muscles are rubbed, and gentle, regulated, voluntary movements are steadily persevered with, and when the union is complete the limb is ready for the act of progression.

As we all know, the persistent inability to use the limb, after union of a fracture of either extremity, is due to the matted and wasted muscles: all this is got rid of if we follow Championnière and Wharton Hood's teaching. Our patients will be ready to use their limb as soon as consolidation of the fracture has taken place. The consolidation takes place as a rule at an earlier period than in a limb treated by complete rest.

Don't fear the pain, it is diminished by the rubbing; and the voluntary movements of the patient are accompanied by little pain if the fracture is steadied by the hands of the surgeon or an attendant, or by the patient himself when he makes the movements. Any pain that takes place is not an obstacle to the movement so long as the pain is not due to the fragments of the bone moving on one another. I am giving these views a fair trial in my practice: they appeal to my psychological instincts; they are in accordance with the treatment that I have used in sprains, dislocations, and in fractures near joints—such as Colles's—for twenty years; and I do not hesitate to ask for them from you careful consideration in all fractures, and, if you see fit to give them a trial in your daily work, I don't think you will be disappointed.

When I think of the value of a free circulation through a part,

I always think of the suction power of a Sprengel pump. Mr. Charles Cathcart has done much for urinary surgery and bladder drainage by applying this principle. May I add that it is also of great value in empyema. How all-present and all-powerful is this principle in our vascular and lymph channels, and in our tissues, which are a sponge: filled with fluid, the fluid in health in constant motion. Any current passing an opening sucks through the aperture the contents of the space beyond: the greater the current the greater the suction.

Let me illustrate my meaning by an example: Why does varicocele occur on the left side? The answer is, because the right spermatic vein opens into the vena cava, the left into the renal vein, and because the suction by the vena cava is infinitely greater than the suction by the renal vein: the greater the current the greater the suction.

So also the rush of blood through the common femoral vein empties the long saphenous vein; so also the short saphenous is emptied by the current through the popliteal vein. Varicose veins are primarily traumatic. A valve is injured in the long saphenous or short saphenous, and the common femoral or popliteal, as the case may be, has too much to do; it cannot empty the superficial vein—the vein gradually dilates, and the neighbouring valves become incompetent; the disease becomes permanent. Varicose veins fill from above, not from below; in other words, the current, if any, is from the deep into the superficial vein; the flow is in an abnormal direction. This, in my opinion, is the way in which rubbing acts in getting rid of the stagnating blood in the paresed vessels in an injured part: it is not by pressing the stagnating blood into the circulating area, but by causing an acceleration of the current in the surrounding veins whereby the blood stasis is gradually removed; therefore the rubbing is to be applied above the seat of injury, and in the direction of the venous and lymphatic flow. So also the regulated voluntary movements increase the blood-flow through the muscles, and thus each vessel in which there is a current acts as a Sprengel pump on the stagnant blood in the neighbourhood. Passive movements can never do this.

In the same way the lymph current acts on the lymph radicles, and they in their turn on the lymph spaces; so also a distended cell element is emptied by the current passing along its walls. At the risk of being tedious, allow me one more illustration to explain my meaning: What is the essential difference between a pond and a river? The water in the pond soaks into the surrounding tissues, the water in the flowing river acts as a Sprengel pump and draws the fluid from the banks; the flow is from the pond, the flow is into the river. This explains the removal of the extravascular effusions from the tissues into the blood and lymph channels. Many look on rubbing as a means of mechanically driving back the effusions into the circulation; no doubt true in part, but not the main truth in estimating the value of rubbing. Look at the boiled lobster-like appearance of a limb after rubbing, an indication of the condition of the deeper parts; look at the accelerated circulation, and we see in that, in my opinion, the main means whereby the stagnant blood in the vessels and the effusions outside the vessels are removed. In a word, the results of every injury are paresis, stagnation, effusion. To get rid of these things (the main obstacles to repair) encourage a free circulation around and through the injured area by rubbing and by regulated voluntary movements, call on the nerves of the part to do their part of the work, avoid passive movements, and don't fear pain due to physiological movements. I have been irresistibly drawn to-night to speak to you of those things which are at the present time uppermost in my mind. I have applied these views to subcutaneous injuries, but we cannot stop there. An aseptic wound is a subcutaneous wound —from the beginning (I happened to be there) this was Lister's aim,—and it must be a question for all of us if open wounds, after all risks of reactionary hemorrhage have passed away, should not be treated on parallel lines. Should we not, after an operation—say, excision of the mamma—encourage gentle voluntary movements of the arm at the shoulder-joint? Should we not encourage the patient to take breathing exercises? Should we not remove the dressings to rub the tissues in the neighbourhood of the wound? After an amputation, should we not rub the limb above the wound, and encourage the patient, after a few days, gently to contract his muscles to move his stump? But I must cease: the prospect leads one on. To dream dreams is pleasant and sometimes profitable; but as in treating a stricture of urethra by gradual dilatation—a form of movement, a variety of rubbing,—when we are in doubt as to whether one more bougie should be passed, it is a good rule to hold your hand; so I will not pursue the subject further, but have, I trust, said enough to make you think about it. As I said when I began, I came to Bristol to give a little and to get much, to hold communion with friends old and new. I have chosen a simple subject—a practical subject—which we can all ponder over. I want, like Goethe, "more light."

Tempted, perhaps, by the fact that there are present, as I am informed, undergraduates of the Bristol College, let me before I sit down take, for a moment, a wider outlook regarding our possibilities and our responsibilities. The younger the man, the greater the possibilities; the older the man, the greater the responsibilities. Age speaking to youth often utters a warning regarding false prophets. I would rather ask you—may I call you my *fellow-students?*—to cultivate true prophets. Six hundred years ago Roger Bacon said that ships would be driven by a machine, with only a pilot on board to steer them; that carriages would be driven by similar means at an incredible rate; and that flying machines would be the favourite method of progression. There are Roger Bacons amongst us to-day, but we do not see them. My prayer for the youth of this country is for clearer vision.

The crop of knowledge has not been reaped. As William Harvey says of the true philosopher, "All we know is still infinitely less than all that remains unknown." We are not the "stubble geese," as was said long ago by a teacher of Anatomy in Edinburgh. William Harvey also says: "Many things may be learned by an old man from a youth, by a person of understanding from one of inferior quality"; and if Harvey could say this, surely we can say it too. Young men see visions; old men dream dreams: both have their value and their inter-actions. From the advances that have been made since I commenced the study of medicine, I am inclined to say that the crop is only sown, and that anyone now beginning his life-work in medicine is to be envied. The world is before him; the riddle has to be read; let him see to it that his possibilities become realities.

I read lately in a novel "there is always the man behind the minister." So there is the man behind the doctor, just as there is the man behind the patient; in other words, the mental factor in disease—a most enticing subject, and, as far as I can judge, one of increasing importance, for which I would ask your careful study and observation. A patient is not simply a pathological entity: his psychological peculiarities are often the outstanding feature in his case. Cultivate the man behind the patient; cultivate the man behind the doctor: by so doing we shall avoid stagnation and paresis. Have a hobby outside your daily work. The danger of stagnation is an ever-present one. The valley of the dry bones of the prophet Ezekiel is to be reckoned with in relation to all three aspects of man's nature—the physical, the intellectual, and the spiritual. It is a danger to the bee, hence a danger to the hive; a danger to the well-being of the leucocyte, hence a danger to the welfare of the universe.

We hear now-a-days a great deal about the consolidation of the British empire. This is not the place to discuss the relative merits of the various prescriptions recommended by many physicians. I will only say that I have heard it said, north of the Border, that if three doctors are called in, the patient is in a dangerous condition; if five take an *active* part in his treatment, his case is hopeless. It is the activity that is the danger. Let us take warning. For my part, I think at present of a greater empire—the Anglo-Saxon empire, with its common language, and, broadly speaking, its common ideals. On its strength depends the welfare of the world.

As I said regarding this, to me, happy visit to your ancient town, you have done me good, strengthening what Walt Whitman calls "the dear love of comrades." And remembering that what is good for the bee is good for the hive, I now say that I know nothing better for progress and happiness, as opposed to selfishness, than the yearly increasing intellectual communion—man meeting man—between this country and

America. "Speak to the boys," as my dear friend the late Dr. David Yandell said to me in the operating theatre in Louisville. My recent African experiences drive home the same lesson. I have now a large number of Boer students in my hospital clinique. Perhaps I may some day see Australasia, and there learn the same lesson as I have already learnt in America, Canada, and South Africa.

The empire of intellect overshadows even the Anglo-Saxon empire. To reap its full possibilities, would that we could go back to the condition when we had a universal language, "when the whole earth was of one language and one speech," when the brotherhood of man was a living reality. We lost it for our sins. A time will come when we will regain it, when we are more able to appreciate its true value; when wars and rumours of wars cease in the land; when we judge not, when we resist not; when we fully understand the full significance of faith, hope, and charity: the greatest of these is charity.

When I speak of faith, hope, and charity, I am at the meeting-place, the trysting-place, between Science and Religion. It has been said that medical men are often materialists. That is not my experience. They may not be theologians, but they are not materialists. There can be no antagonism between true science and true religion: they clash only when they are false. Their present antagonism is only another word for our ignorance. Our very imperfections necessitate antagonism.

As time goes on we shall see more clearly; the mists will gradually disappear. Towards that end—the lifting of the mist—let us, in the meantime, each add his little pebble to the cairn of human knowledge; and let us always remember that "little drops of water," "little grains of sand," "little deeds of kindness," are all-powerful in the unfolding of our race.

We cannot all be in the front rank. The rear-guard is often the salvation of the column. The universe has need of all men. It seems to me a sound, and certainly it is to me a comforting, philosophy which says to each of us:—

> "Do what you can, being what you are.
> Shine like a glow-worm if you cannot be a star.
> Work like a pulley if you cannot be a crane.
> Be a wheel-greaser if you cannot drive a train."

